# HIV seropositivity, patterns, and clinico-epidemiological profile of sexually transmitted infection patients attending the Suraksha Clinic of a tertiary care public hospital in southern Rajasthan, India—a cross-sectional study

**DOI:** 10.1038/s41598-025-25015-2

**Published:** 2025-11-20

**Authors:** Shikha Mehta, Rajath Rao, Sharad Mehta, Keerti Singh

**Affiliations:** 1 Department of Community Medicine, RNT Medical College, Udaipur, India; 2 Department of Community Medicine, Kasturba Medical College Mangalore, Manipal Academy of Higher Education, Manipal, India; 3 Department of Dermatology and Venerology, RNT Medical College, Udaipur, India

**Keywords:** Sexually transmitted infections, HIV, Suraksha Clinic, Sexual and reproductive health, National AIDS Control Organization, Diseases, Health care, Medical research, Microbiology, Risk factors

## Abstract

The current study aimed to determine the clinical-epidemiological profile, patterns, and HIV seropositivity among STI patients attending the Suraksha Clinic by the National AIDS Control Organization in southern Rajasthan, India. This cross-sectional study was performed among 300 STI patients via a questionnaire. The proportions of STI patients with various clinical-epidemiological backgrounds were expressed as frequencies and percentages. The associations between the sex distributions of the clinical parameters of STI patients were assessed via the chi-square test. Among the 300 STI patients, nearly three-fourths (74%) were males. Only 85 (28.3%) used condoms. Nearly half of them (51%) had their first sexual contact at approximately 15–19 years of age, and more than half (52.7%) had more than one sexual partner. The most common STIs were herpes genitalis (56.8%) in males and vaginal discharge (58.9%) in females. Overall, HIV seropositivity was observed in 7.7% (95% CI: 5.2–11.2%) of patients. HIV seropositivity was more common among the bridge population (17.2%) than among the other populations (4.2%). Only one out of four STI patients used condoms. One out of two had more than one sexual partner. Almost one in ten STI patients were HIV seropositive. The bridge population were more likely to be HIV seropositive than the other populations were.

**Supplementary Information:**

The online version contains supplementary material available at 10.1038/s41598-025-25015-2.

## Introduction

Sexually transmitted infections (STIs) are a major public health problem worldwide, including in India. Every day, more than 1 million STIs occur, which are mostly asymptomatic. Multiple pathogens are involved in the causation of sexually transmitted diseases (STDs), which are caused mainly by bacteria and viruses^1^. STIs are disproportionately prevalent in low- and middle-income (LMIC) countries, with 75–85% of new cases occurring in these places^2^. The common STIs are syphilis, chlamydia, trichomoniasis, gonorrhea, HIV, hepatitis B, human papillomavirus, and herpes simplex virus. One in every four new cases is due to chlamydia, gonorrhea, syphilis, or trichomoniasis. The symptoms of these infections range from discharge from the urethra, discharge from the vagina, burning micturition, lower abdominal pain, genital ulcers, etc. The mode of transmission is mainly sexual contact (anal, oral, or vaginal), which can also be transmitted during pregnancy, delivery, or lactation^3^. Untreated STIs can result in many complications, such as pelvic inflammatory diseases, ectopic pregnancy, and infertility. Mother-to-child transmission of STIs can result in stillbirth, neonatal death, low birth weight and prematurity, sepsis, neonatal conjunctivitis, and congenital deformities^1^. Added to this, rapidly increasing antimicrobial resistance is a growing threat to the treatment of gonorrhea^4^. In addition to complications from such infections, other concerns, such as psychosocial burdens, prevail among affected individuals. The main concern is that STI promotes human immunodeficiency virus (HIV) transmission by increasing HIV infection and susceptibility^5^. In India, the disease prevalence is estimated to be 6%. According to the NFHS-5 (2019-21), 12% of women aged 15–49 years who have ever had sex and 9% of men aged 15–49 years who have ever had sex reported having an STI and/or symptoms of an STI in the past 12 months^6^. The estimates also indicate that approximately 40% of women have RTIs/STIs at any given point in time, but only 1% complete the full treatment of both partners^7^.

In India, through its network of 1160 designated STI/RTI clinics, which are usually situated at government health care facilities at the district level, medical colleges and above provide free standardized STI/RTI services. These clinics have been branded as ‘Suraksha Clinics’ and provide sexual and reproductive health services^8^. Treating STIs reduces the prevalence and breaks the chain of transmission in the community and is therefore one of the most effective forms of prevention in the absence of a vaccine^9^. A systematic, regional, periodic synopsis of the prevalence of STDs among Suraksha clinic attendees would not only help to study the changing trends of STDs but also assess the effectiveness of control programmes. Early diagnosis and STI case management are cornerstones of STI control, as they reduce the prevalence and break the chain of transmission in the community. In this context, this study aimed to determine the pattern, clinical-epidemiological profile and HIV seropositivity among STI patients as a primary objective and to determine the association of various STI patterns with clinico-epidemiological variables among patients attending the Suraksha Clinic of a government medical college and hospital in southern Rajasthan as a secondary objective.

## Materials and methods

### Study design and duration

A facility-based cross-sectional study was performed for a duration of one year (January to December 2019).

### Study setting

This study was carried out in the Department of Dermatology and Venerology in association with the Department of Community Medicine of a district Government medical college and hospital in southern Rajasthan. This hospital is a tertiary health care facility catering to the population ofUdaipur city and the neighboring southern districts of Rajasthan with a 1000-bed capacity. The Department of Dermatology and Venerology, established in 1960, has been providing tertiary dermatology care and venerology care in its Suraksha Clinic. The Suraksha Clinic screens and treats approximately 25–30 suspects and cases of STI every day.

**Fig. 1:**
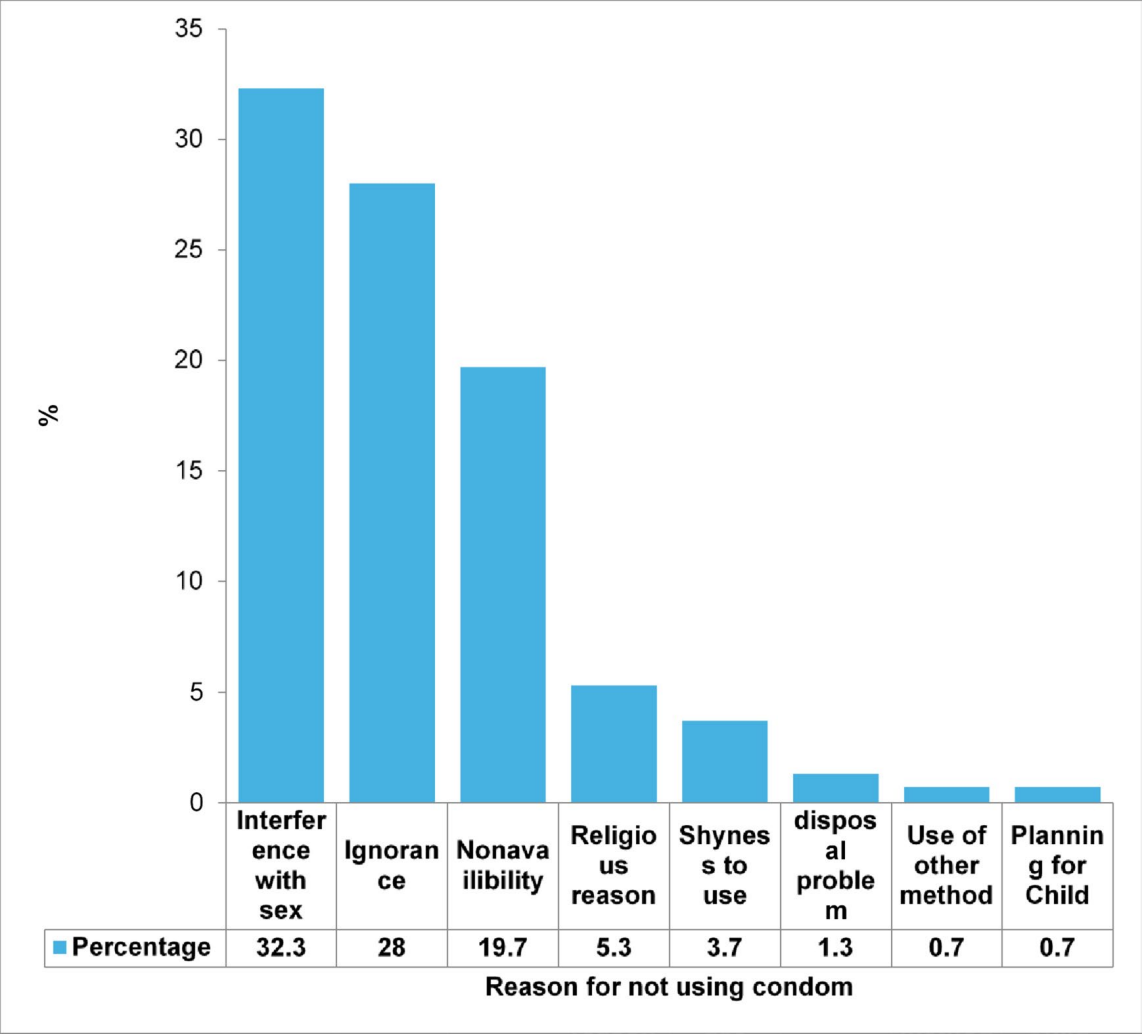
Reasons for not using the condom among STI patients [*n* = 275 (Never users = 215, Occasional users = 60)]

**Fig. 2:**
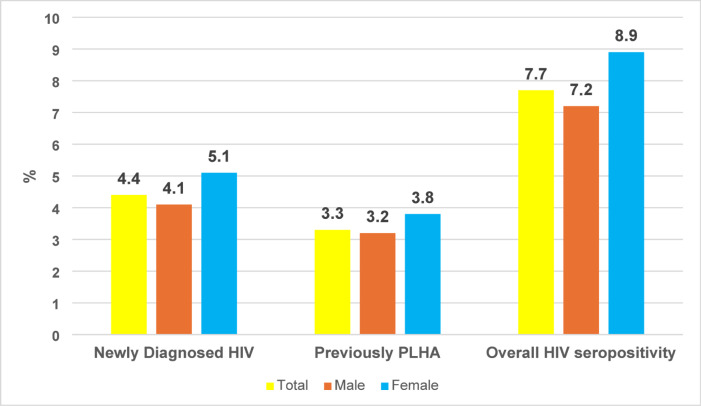
Gender distribution of HIV seropositivity among STI patients attending the Suraksha Clinic (*N* = 300).

### Study population

This study was carried out among STI patients attending the Suraksha Clinic. All patients with STIs attending the Suraksha Clinic, including adolescents, were included in the study. STI patients who were unable to comprehend the interview, those with self-reported psychiatric ailments or any serious illness or comorbidities, and those who did not provide informed written consent to participate in the study were excluded from the study.

### Sample size and sampling technique

Considering that the HIV seropositivity among STI patients is approximately 34.5%^10^, we needed a minimum sample size of 290 at 95% CI, 80% power, and finite population adjustment of 1800 STI cases attending the Suraksha Clinic for the previous year. The final sample size was rounded to 300 STI cases to determine the outcome. The sample size was calculated via Statulator, an online software for sample size calculation^11^.

We adopted a consecutive sampling technique, a non-probability sampling method, to select patients who were coming to the Suraksha Clinic after meeting the inclusion and exclusion criteria.

### Study tools and techniques

A pretested, structured study tool was used for the data collection. The study tool was initially written in English and then translated to the local language (Hindi) and back-translated to English with the help of medical social workers of the Department of Community Medicine. The study tool consisted of various sections. Section A consisted of sociodemographic variables such as age, sex, education, occupation, religion, monthly family income, socioeconomic class, type of family, marital status, substance use, whether they belong to high-risk groups, and the bridge population. Bridge populations comprise people who, through proximity to high-risk groups, are at risk of contracting HIV. Quite often, they are clients or partners of male and female sex workers. Truckers and migrant laborers are major bridge populations^12^. Section B consisted of items related to sexual history, such as chief complaints, onset and duration of disease, history of sexual exposure, last sexual activity, age of first sexual contact, type of sexual behavior, and nature of the contraceptives used. In section C, the pattern of STI, such as the number of lesions, type of lesion, and type of discharge, is described. Section D contains investigations such as Gram staining findings, KOH tests, Tzank smears, WHIFF tests, wet mounts, VDRL tests, HIV tests, urine examinations, blood sugar level tests, and final diagnoses on the basis of all these findings. The study tool was administered in the local language through face‒to-face interviews by the investigators in the presence of a dermatologist and an STI counsellor. Privacy and confidentiality were maintained throughout the study. The collected data were entered into MS Excel. All the laboratory investigations were performed as per the standard protocol and as a part of the SOP to be performed at the Suraksha Clinic as issued by the NACO.

### Statistical analysis plan

The data analysis was performed via IBM SPSS V.20.0. A descriptive analysis was carried out to determine the clinical‒epidemiological profile and pattern of STI patients attending the Suraksha Clinic. Sociodemographic variables such as education, occupation, marital status, and residence; epidemiological variables such as a history of sexual exposure/contact, the use of barrier methods of contraception, the reasons for using and not using condoms, the type of high-risk group, the bridge population, substance use, circumcision, the age of the first sexual act, sexuality, and the number of sexual partners; and clinical findings such as the number of genital lesions, the nature of the lesion, infective agents, and the diagnosis were expressed as frequencies and percentages. The proportion of HIV seropositive individuals is expressed as percentages with 95% CIs. To determine the associations between various sociodemographics and epidemiological variables and HIV seropositivity, a chi-square test of associations was used. All statistical significance was attributed to a p value < 0.05.

### Ethical considerations

The study was approved by the institutional ethics committees of RNT Medical College and MB Government Hospital, Udaipur (Ref: RNT/Stat/IEC/2019/405). Written informed consent was obtained from all the patients. The patient information was kept confidential, and all identifiers and enablers were removed. The study followed the Declaration of Helsinki.

## Results

### Sociodemographic details of the STI patients

Among the 300 STI patients attending the Suraksha Clinic, most (206, 68.7%) were 18–35 years of age, approximately three-fourths (222, 74%) were males, and half (153, 51%) were from urban areas. Nearly 207 (69%) were married, and the maximum (288, 96%) belonged to the Hindu religion. Only 31 (10.3%) were illiterate, 120 (40%) were clerical/shop owners/farmers by occupation, and more than half (166, 55.4%) were in the socioeconomic class of class 4 or above. Approximately 11 (3.6%) were homosexual, and 63 (28.4%) belonged to a bridge population. Approximately 80 (26.7%) used either form of tobacco (Table 1).

**Table 1:** Sociodemographic details of the patients attending the Suraksha clinic (*N* = 300).

Variables	Category	*n* (%)
Age (in years)	< 18	39 (13)
	18–35	206 (68.7)
	36–55	52 (17.3)
	> 55	3 (1)
Gender	Females	78 (26)
	Males	222 (74)
Residence	Urban	153 (51)
	Rural	147 (49)
Marital status	Married	207(69)
	Unmarried	80(26.7)
	Divorced	4(1.3)
	Widow	7(2.3)
	Separated	2 (0.7)
Religion	Hindu	288 (96)
	Muslim	6 (2)
	Jain	3 (1)
	Others*	3 (1)
Education status	Illiterate	31(10.3)
	Primary	87(29.0)
	Secondary	55(18.3)
	Senior Secondary	15(5.0)
	Graduate	70(23.3)
	Postgraduate	39(13.0)
	Professional	3(1.0)
Occupation	Semi-professional/professional	3 (1.0)
	Clerical, shop-owner, farmer	120.0 (40)
	Skilled/semiskilled worker	68 (22.7)
	Unskilled worker	53 (17.7)
	Unemployed	56 (18.6)
Socioeconomic status**	Class 1	20 (6.7)
	Class 2	39 (13.0)
	Class 3	75 (25.0)
	Class 4	119 (39.7)
	Class 5	47 (15.7)
High-risk groups	MSM	11 (3.6)
	IV drug users	2 (0.7)
	Partners of CSW	16 (5.4)
Bridge Population***	Males (*n* = 222)	63 (28.4)
	Male partners of female patients (*n* = 78)	19 (24.4)
Substance use	None	174(58)
	Alcohol	64(21.3)
	Tobacco (smoke form)	36(12.0)
	Tobacco (smokeless)	44(14.7)
	IV drug	2(0.7)
Comorbidity	Diabetes mellitus	18 (6)
	Hypertension	8 (2.7)
	Thyroid disorder	2 (0.7)
	Others (CVS, migraine)	7 (2.3)
	None	265 (88.3)

*Others included Christians, Sikhs; **by Modified BG Prasad classification; ***includes truck drivers and migrant workers; MSM-Males having sex with males; CSW- Commercial sex workers.

### Epidemiological details of the STI patients

Among the 222 males, only 7 (3.2%) were circumcised. Nearly half of the participants (*n* = 139, 46.3%) used either type of contraceptive. Only 85 (28.3%) used condoms, and 25 (8.3%) used condoms regularly. Nearly 33% of patients felt that condoms interfered with their sexual pleasure and did not use it (Fig. 1). Nearly half of them, 153 (51%), had their first sexual contact at approximately 15–19 years of age, and a maximum of 288 (96%) engaged in penovaginal intercourse. More than half of the participants (158, 52.7%) had more than one sexual partner, and 167 (55.6%) had regular sexual partners. Only 55 (18.4%) had used condoms in their last coital activity (Table 2).

**Table 2:** Epidemiological details of the patients attending the Suraksha clinic (*N* = 300).

Variables	Category	*n* (%)
Circumcision	Male patients (*n* = 222)	7 (3.2)
	Male partners of female patients (*n* = 78)	1 (1.3)
**Any contraceptive usage**		**139 (46.3)**
Types of contraceptives (*n* = 139)	Condom	85(28.3)
	Sterilization	23(7.7)
	Oral pills	11(3.7)
	Copper T	10(3.0)
	Injectables	2(0.4)
	Rhythm method (Natural)	8(2.7)
Frequency of condom usage	Never	215 (71.7)
	Occasional	60 (20)
	Regular	25 (8.3)
Age of first sexual contact	10–14 years	12(4)
	15-19 years	153(51)
	20-24 years	111(37)
	25-29 years	21(7)
	> 30 years	3(1)
Type of sexuality	Heterosexual	289 (96.4)
	Homosexual	7 (2.3)
	Bisexual	4 (1.3)
Type of sexual behavior	Oral	0
	Vaginal	288(96)
	Anal	8(2.6)
	Mixed	4(1.4)
Number of partners	Single partner	142(47.3)
	Multiple partner	158(52.7)
Type of partner	Occasional	61(20.4)
	Regular	167(55.6)
	Both	72(24)
History of condom usage in last coitus	Used	55 (18.4)

### Clinical profile of the STI patients

Among 300 STI patients, 261 (87%) had a single lesion in the genital tract. Nearly half of the patients (149 (49.7%)) had ulcerative lesions. The majority, 235 (78.4%), had a viral agent as the causative organism. The overall most common STI was herpes genitalis (158, 52.7%), followed by vaginal discharge (46, 15.3%) and syphilis (13.3%). Among males, herpes genitalis was most common (*n* = 126, 56.8%), and among females, vaginal discharge was most common (*n* = 46, 58.9%). More than half of the patients (159, 53%) had vesicular lesions, followed by papules (72, 24%). The overall most common presenting symptom was genital ulcers (62%), followed by burning urination (34.7%) and itching in the genital area (25.3%). Among males, the most common symptom was genital ulcers (*n* = 148, 66.7%), followed by burning urination (*n* = 70, 31.5%). Among females, the most common symptom was itching in the genitals (*n* = 49, 62.8%), followed by discharge (*n* = 46, 58.9%). Nearly one-third (109, 36.4%) had symptoms between 10 and 30 days in duration (Table 3).

**Table 3:** Gender distribution of the clinical profile of STI patients attending the Suraksha clinic (*n* = 300).

Variables	Category	*n* (%)	Male, *n* = 222 (%)	Female, *n* = 78 (%)	*P* value
Number of lesions in the genitals*	Single	261(87)	207(93.2)	54(69.2)	< 0.001
	Multiple	39(13)	15(6.8)	24(30.8)	
Nature of lesion*	Ulcerative	149(49.7)	129(58.1)	20(25.6)	< 0.001
	Non-ulcerative	125(41.7)	82(36.9)	43(55.1)	
	Mixed	26(8.6)	11(4.9)	15(19.2)	
Type of agent ^a^	Viral*	235 (78.4)	183 (82.4)	52 (17.4)	0.006
	Bacterial	52 (17.4)	32 (14.4)	18 (23)	0.1
	Fungal*	57 (19)	21 (9.4)	36 (46.1)	< 0.001
Pattern of STI ^a^	Herpes genitalis*	158(52.7)	126(56.8)	32(41.0)	0.02
	Syphilis	40(13.3)	32(14.4)	8(10.2)	0.4
	Vaginal discharge	46(15.3)	0	46(58.9)	-
	Urethral discharge	2(0.7)	2(0.9)	0	-
	Molluscum*	43(14.3)	26(11.7)	17(21.7)	0.04
	Wart*	34(11.3)	31(14.0)	3(3.8)	0.02
	HIV	23(7.7)	16(7.2)	7(9)	0.7
	Balanoposthitis	21(7.0)	21(9.5)	0	-
Type of lesion ^a^	Papule	72(24.0)	51(23)	21(26.9)	0.5
	Vesicle*	159(53.0)	126(56.8)	33(42.3)	0.03
	Pustule	3(1.0)	2(0.9)	1(1.3)	0.7
	Ulcer	29(9.7)	22(9.9)	7(9.0)	0.8
	Warts*	34(11.3)	31(14)	3(3.8)	0.02
	Discharge*	48(16.0)	2(0.9)	46(58.9)	< 0.001
Symptoms ^a^	Genital discharge*	48(16)	2(0.9)	46(58.9)	< 0.001
	Itching in genitals*	76(25.3)	27(12.1)	49(62.8)	< 0.001
	Burning micturition	104(34.7)	70(31.5)	34(43.6)	0.07
	Genital ulcer*	186(62)	148(66.7)	38(48.1)	0.007
	Inguinal/scrotal swelling*	21(7)	20(9.0)	1(1.2)	0.02
	Abnormal bleeding	20(6.7)	0(0)	20(25.6)	-
	Painful coitus*	35(11.7)	15(6.7)	20(25.6)	< 0.001
	Wart in genitals*	34(11.3)	31(14)	3(3.8)	0.02
	Lower abdominal pain	2(2.5)	0(0)	2(2.5)	-
Duration of symptoms	< 10 days	132(44)	101(45.5)	31(39.7)	0.08
	10–30 days	109(36.4)	84(37.8)	25(32.1)	
	> 30 days	59(19.6)	37(16.7)	22(28.2)	

^a^Multiple response items; *statistically significant (*p* < 0.05) by chi-square test of association/Fischer exact test.

Compared with females, males presented with more single genital lesions (males: 93.2% vs. females: 69.2%, *p* < 0.001), ulcerative lesions (males: 58.1% vs. females: 25.6%, *p* < 0.001), viral causative agents (males: 82.4% vs. females: 17.4%, *p* = 0.006), vesicular lesions (males: 56.8% vs. females: 42.3%, *p* = 0.03), and warts (males: 14% vs. females: 3.8%, *p* = 0.02), and these differences were statistically significant. Similarly, females presented with more lesions of fungal origin (females: 46.1% vs. males: 9.4%, *p* < 0.001), molluscum contagiosum (females: 21.7% vs. males: 11.7%, *p* = 0.04), discharge from the lesion (females: 58.9% vs. males: 0.9%, *p* < 0.001), itching in the genitals as a main complaint (females: 62.8% vs. males: 12.1%, *p* < 0.001), and painful coitus (females: 25.6% vs. males: 6.7%, *p* < 0.001) than their counterparts did, and these differences were statistically significant (Table 3).

### HIV seropositivity among STI patients

Among the 300 STI patients, 7.7% (*n* = 23, 95% CI: 5.2–11.2%) were HIV seropositive, and 13 (4.4%, 95% CI: 2.5–7.2%) were newly diagnosed with HIV. There were no statistically significant differences in HIV seropositivity between males (*n* = 16, 7.2%) and females (*n* = 7, 8.9%) (*p* = 0.6) (Fig. 2). Additionally, we observed that the distribution of HIV-positive patients was greater among the bridge population (*n* = 14, 17%) than among other STI patients (*n* = 9, 4.2%), and this difference in proportion was statistically significant (*p* < 0.001) (Table 4).

**Table 4:** Association of HIV seropositivity among various types of STI patients (*n* = 300).

HIV status	Bridge population (*n* = 82), %	Other STI patients (*n* = 218), %	Total (*n* = 300), %
HIV positive	14(17.0)	9(4.2)	23(7.7)
HIV negative	68(82.9)	209(95.8)	277(92.3)

Chi-square value (df), p value = 14.1 (1), < 0.001.

### Association between epidemiological variables and patterns of stis among the patients attending the Suraksha Clinic

On univariate analysis, it was found that the age of the STI patients, marital status, number of sexual partners, previous history of STI, and age at first coitus were associated with various patterns of STI. Most of the STIs, except urethral discharge, were common in the 18–35 years age group compared to others (*p* = 0.01). Similarly, married individuals had most of the STIs compared to others. (*p* = 0.007) Most STIs were common in STI patients with multiple sexual partners compared to others, except for molluscum contagiosum, which was common in STI patients with single partners. (*p* < 0.001) Also, STIs were common among individuals with a previous history of STI (*P* = 0.04) and a younger age (10–14/15–19 years) of first sexual activity/coitus compared to others. (*p* < 0.001) [Supplementary Table S1].

## Discussion

The present cross-sectional study in Udaipur, Rajasthan, aimed to study the sociodemographic profile, the pattern of STIs, and HIV seropositivity among STI patients attending the Suraksha Clinic.

Our study reported 222 (74%) males with a male‒female ratio of 2.84:1. Similar studies conducted by Suvirya et al.^5^, Devi et al.^10^, and Sharma R et al.^13^ reported a greater number of STIs in males than in females, which supports the findings of our current study. In a study by Vora R et al.^14^, females outnumbered males (1:0.2), as many patients were referred from the gynecology department. The most likely cause of the low proportion of STIs in females in comparison with males may be the low reporting of females to the STI clinic, the asymptomatic nature of STDs in females, a lack of knowledge, and gender inequality^4^. The majority of male and female patients were adults (≥ 18 years of age). The most common age group affected was 18 to 35 years (71%). The same result was observed in various previous Indian studies^15,16^. These findings indicate that 18 to 35-year-olds were comparatively more active and therefore more susceptible to STIs than their counterparts were. Additionally, this group is the most productive age group. Education and occupation lead to migration, and adventurousness/curiosity to try new things increases the risk of STIs. Our study reported equal representation from urban and rural areas. In contrast, a study conducted by Nyati A. et al. in a tertiary care centre in Rajasthan reported that two-thirds of the population resided in urban areas^17^. The low attendance of the rural population at STI clinics signifies a lack of awareness and knowledge about STIs and a disparity in access to healthcare services. In our study, the majority of patients were married (69%), which is supported by other Indian studies^13,14^. Factors such as infidelity and a lack of condom use among married people add burdens to STIs. The maximum number of patients (95% males, 98.7% females) belonged to the Hindu community. Similarly, in the study by Suvirya S et al., the majority belonged to the Hindu community^5^. Since Muslim males have their circumcision done at an early age, there are low chances of STI among this community^17^. In our study, approximately 10% of the STI patients belonged to the high-risk group, and nearly one-fourth (27.4%) belonged to the bridge population (drivers and migrant laborers). The truck drivers and the migrant laborers stay away from home for long periods, and because of this, they meet multiple partners and commercial sex workers. This finding highlights the need for targeted interventions for these groups of people.

In our study, nearly half (48.6%) of the patients had a history of substance use. The majority were alcoholics (21.3%). A greater proportion of alcoholics with STIs was reported in other Indian studies^18–20^. Under the influence of alcohol, there is a loss of control over sexual behaviors and an increase in the desire for sexual intercourse. The mean age of sexual debut was 19.7 years in males and 18.4 years in females. More than half (51%) had their first coital debut between 15 and 19 years. This may predispose patients to a greater risk of contracting HIV. This finding was comparable to that of the study conducted by Maheswari SU et al., where the mean age of coital debut was 21.1 years in males and 18.6 years in females^21^. The reason for the lower age of coital debut in females in our country is likely early marriage among females in India.

Among the 300 patients, nearly half (49.7%) had ulcerative STIs, and this was more common in males (58%). Similarly, in a study by Devi S A et al., a male predominance was observed^10^. Viral infections (78.4%) were almost 5 times more common than bacterial infections. Herpes genitalis (52.7%) was the most common viral infection, and syphilis (13%) was the most common bacterial infection. Similar results were reported in other Indian studies, such as those of Setia MS et al.^22^, Mendiratta V et al.^23^, and Narayanan Bet al.^24^. This increase in viral STIs may be due to increased self-medication or antibiotic use, which can treat some bacterial STIs, treatment of bacterial STIs at the primary care level, and an actual change in the pattern of STIs. The roles of antibiotic resistance and changes in sexual behaviour need to be explored.

Our study reported an HIV seropositivity of approximately 8%. Other studies reported HIV seropositivity rates of approximately 10–35%^10,16,25–29^. Our study reported greater HIV seropositivity among female STI patients (8.9%) than among male patients (7.2%). In contrast, in a study by Devi S A et al., more male patients than female patients were HIV positive^10^. Biologically, females are more vulnerable to HIV infection due to the large surface area of the genital tract exposed, and factors such as gender-based disparities in accessing healthcare, ignorance, and neglect add to high HIV seropositivity. In our study, a higher percentage of HIV-positive patients (17%) than other STI patients (4.2%) were found in the bridge population. Similarly, high HIV seropositivity among the bridge population (56.86%) was observed in a study conducted by DM Thappa in Pondicherry^27^. This demands focused prevention efforts, including improved access to condoms, HIV testing services, and antiretroviral therapy including pre-exposure prophylaxis. (PrEP)

A few strengths of the study are that it included a significant number of patients representing the sample of STI patients. The study detailed the essential sociodemographic, behavioral, and clinical information of the STI patients. The study highlights high-risk groups such as MSM and bridge populations, informing targeted interventions. This study is not without limitations. The few limitations are the cross-sectional nature of the study, self-reported data on sexual history, and potential selection bias in approaching patients seeking healthcare in facilities. Also, adding confirmatory tests for all the conditions will add more reliability to the study. A multivariate analysis of epidemiological data and STI data would have improved the generalizability of the article.

## Conclusion and recommendations

Our study highlights the significant burden of STIs among the adult population attending the Suraksha Clinic, which is predominant among males aged 18–35 years. The equal distribution of STI patients across urban‒rural areas highlights the pervasive nature of the problem. The high proportion of married patients suggests the need for comprehensive sexual health education and couple counseling programs within the community. The strong association between substance use and STI emphasizes the importance of integrated preventive strategies covering both issues. Nearly one in ten STI patients are HIV seropositive, with more females being HIV positive, underscoring the need for gender-specific prevention and care initiatives.

There is an urgent need for strengthened STI prevention and control programs, including Suraksha clinics, and more targeted interventions for high-risk populations. Continuous training in syndromic management and the establishment of regular supportive supervision are necessary for both general practitioners and specialists. Government and program planners should consider the concerns expressed by patients regarding health service-related issues such as the availability of medicines, confidentiality issues, stigmatization, and harassment from staff. Individuals suffering from STI should be made aware that they must seek treatment promptly and refrain from sexual activity until they have been effectively treated.

### What is already known about this topic

- Sexually transmitted infections are a significant public health issue, and HIV is a major health concern globally. In 2023, approximately 1.2 million adults aged > 15 years acquired HIV infection.
- Illiteracy, promiscuity, and stigma associated with STI and HIV care were factors associated with the increasing burden of STI, including HIV.

### What does this study add?

- This study from the largest state of India highlights the increasing burden of STIs and more patients acquiring HIV seropositivity with various demographic and behavioral factors, including high-risk behaviors such as early age of initiation of first sexual activity and multiple sexual partners, which are significant risk factors for STIs and HIV.
- This study highlights the nature and pattern of STIs among patients seeking health care in a district public hospital. Identifying the most common sex-specific STIs, overall HIV seropositivity among these STI patients, and how the bridge population has a relatively high risk of HIV infection could play crucial roles in focused HIV prevention efforts.
- This study highlights the need for enhanced STI care and HIV prevention, improved STI and STI care and the need to strengthen existing surveillance systems to monitor trends in STI and HIV incidence in this part of India and can inform evidence-based interventions to address these existing public health challenges.

## Supplementary Information

Below is the link to the electronic supplementary material.

Supplementary Material 1

## Data Availability

The data are available upon request to the corresponding author via email.
