# Supplementary material for: HIV seropositivity, patterns, and clinico-epidemiological profile of sexually transmitted infection patients attending the Suraksha Clinic of a tertiary care public hospital in southern Rajasthan, India—a cross-sectional study
Source: Sci Rep. 2025 Nov 20;15:41027. doi: 10.1038/s41598-025-25015-2 (PMC12635183; doi:10.1038/s41598-025-25015-2)
Supplement: Supplementary file 1 — Supplementary Material 1 [file 41598_2025_25015_MOESM1_ESM.docx]

**Supplementary Materials**

**Table S1. Association of patterns of STI with epidemiological and STI-related variables (N=300)**

| **Variables** | **Category** | **Herpes Genitalis (n=158)** | **Syphilis (n=40)** | **Vaginal Discharge (n=46)** | **Urethral Discharge (n=2)** | **Molluscum (n=43)** | **Wart (n=34)** | **HIV (n=13)** | **Balanoposthitis (n=21)** | **PLHA (n=10)** | **p-value (by chi-square/Fisher's exact test)** |
| --- | --- | --- | --- | --- | --- | --- | --- | --- | --- | --- | --- |
| **Age group of STI patients (years)** | **<18** | 19(12.0%) | 6(15.0% | 5(10.9%) | 1(50%) | 2(4.7%) | 10(29.4%) | 1(7.7%) | 2(9.5%) | 0(0%) | **0.01*** |
|  | **18-35** | 110(69.6%) | 29(72.5%) | 37(80.4%) | 0(0%) | 36(83.7%) | 16(47.1%) | 9(69.2%) | 14(66.7%) | 6(60%) |  |
|  | **36-55** | 28(17.7%) | 5(12.5%) | 4(8.7%) | 1(50%) | 5(11.6%) | 6(17.6%) | 3(23.1%) | 5(23.8%) | 4(40%) |  |
|  | **>55** | 1(0.6%) | 0(0%) | 0(0%) | 0(0%) | 0(0%) | 2(5.9%) | 0(0%) | 0(0%) | 0(0%) |  |
| **Marital status** | **Married** | 110 (69.6%) | 24 (60%) | 40(86.9%) | 1(50%) | 36(83.7%) | 13(38.2%) | 11(84.6%) | 17 (80.9%) | 8(80%) | **0.007*** |
|  | **Unmarried** | 43(27.2%) | 11(27.5%) | 4(8.6 %) | 1(50%) | 7(16.2%) | 17(50%) | 1(7.6%) | 4(19.0%) | 0(0%) |  |
|  | **Divorce** | 1(0.63%) | 1(0.63%) | 0(0%) | 0(0%) | 0(0%) | 2(5.8%) | 1(7.6%) | 0(0%) | 1(10%) |  |
|  | **Widow** | 3(1.8%) | 3(7.5%) | 1(2.1%) | 0(0%) | 0(0%) | 2(5.8%) | 0(0%) | 0(0%) | 1(10%) |  |
|  | **Separated** | 1(0.63%) | 1(2.5%) | 1(2.1%) | 0(0%) | 0(0%) | 0(0%) | 0(0%) | 0(0%) | 0(0%) |  |
| **Area of residence** | Urban | 84(53.1%) | 14(35%) | 17(36.9%) | 0(0%) | 25(58.2%) | 16(47.0%) | 7(53.8%) | 14(66.6%) | 2(20%) | **0.07** |
|  | Rural | 74(46.8%) | 26(65%) | 27(58.6%) | 2(100%) | 18(41.8%) | 18(52.9%) | 6(46.1%) | 7(33.3%) | 8(80%) |  |
| **Number of sexual partners** | **Single Partner** | 64(40.5%) | 18(45.0%) | 34(73.9%) | 0(0%) | 27(62.7%) | 10(29.4%) | 5(38.4%) | 10(47.6%) | 4(40%) | **<0.001*** |
|  | **Multiple partner (%)** | 94(59.5%) | 22(55%) | 12(26.0%) | 2(1000%) | 16(37.2%) | 24(70.5%) | 8(61.5%) | 11(52.3%) | 6(60%) |  |
| **Previous history of STI** | **Present** | 75(47.4%) | 14(35%) | 19(41.3%) | 0(0%) | 11(25.5%) | 10(29.4%) | 3(23%) | 14(66.6%) | 9(90%) | **0.04*** |
|  | **Absent** | 83(52.5%) | 26(65%) | 27(58.6%) | 2(100%) | 32(74.4%) | 24(70.5%) | 10(76.9%) | 7(33.3%) | 1(10%) |  |
| **Age at first coitus (Years)** | 10-14 | 6(3.7%) | 2(5%) | 2(4.3%) | 0(0%) | 2(4.6%) | 2(5.8%) | 0(0%) | 1(4.7%) | 0(0%) | **<0.001*** |
|  | 15-19 | 82(51.8%) | 21(52.5%) | 32(69.5%) | 1(50%) | 16(37.2%) | 20(58.8%) | 8(61.5%) | 6(28.5%) | 8(80%) |  |
|  | 20-24 | 58(36.7%) | 17(42.5%) | 12(26.0%) | 1(50%) | 21(48.8%) | 8(23.5%) | 3(23.0%) | 9(42.8%) | 2(20%) |  |
|  | 25-29 | 10(6.32%) | 0(0%) | 0(0%) | 0(0%) | 4(0.9%) | 3(8.8%) | 2(15.3%) | 5(23.8%) | 0(0%) |  |
|  | >30 | 2(1.2%) | 0(0%) | 0(0%) | 0(0%) | 0(0%) | 1(2.9%) | 0(0%) | 0(0%) | 0(0%) |  |

**NB: PLHA-People Living With HIV**
